# An attempt to improve Ferreira-Junior model concerning the anti-inflammatory action of whole-body cryotherapy after exercise induced muscular damage (EIMD)

**DOI:** 10.3389/fphys.2015.00035

**Published:** 2015-02-12

**Authors:** Benoit M. Dugué

**Affiliations:** Laboratoire Mobilité Vieillissement Exercice, Faculty of Sport Sciences, University of PoitiersPoitiers, France

**Keywords:** cryotherapy, cryostimulation, sICAM-1, exercise, exercise induced muscular damage

Recently, an interesting model trying to explain the anti-inflammatory action of whole body cryotherapy (WBC) after exercise induced muscular damage (EIMD) was published by Ferreira Junior et al. in Frontiers in Physiology (Ferreira-Junior et al., 2014). The authors have suggested that soluble intercellular adhesion molecule-1 (sICAM-1) could be one of the main influencing compounds that could regulate the cryotherapy anti-inflammatory response. According to the authors, after EIMD sarcomeres are disrupted leukocytes (neutrophils, monocytes, and lymphocytes) are mobilized to the injured tissue via sICAM-1. Afterward, pro-inflammatory cytokines and reactive oxygen species are produced in muscle by leukocytes. Together, leukocytes, pro-inflammatory cytokines, and reactive oxygen species cause intramuscular degradation, which amplifies the initial muscle damage.

The authors suggested that a drop in core temperature induced by WBC would likely cause constriction of local arterioles and venules and lower the amount of leukocytes arriving to the exercise-induced muscular inflammation. Moreover, WBC may hasten the recovery from EIMD by reducing sICAM-1 and therefore limiting the migration of leukocytes from blood circulation to the damaged tissues.

The first part of the explanation concerning the WBC-induced vasoconstriction is understandable though a drop in core temperature is not always clear (no changes in core temperature has been observed after 2 min exposure at −110°C Westerlund et al., 2003). However, concerning the second part of the explanation dealing with a lower concentration of circulating sICAM-1. I would argue that an increase in the blood concentration of this analyte might paradoxically occur.

Intercellular adhesion molecule-1 is a widely distributed adhesion factor present on the surfaces of endothelial cells and leukocytes. This protein mediates adhesion and transmigration of leukocytes through the endothelium. Surface expressed ICAM-1 is apparently shed from the cells and then circulates as soluble ICAM-1 (sICAM-1) although the full mechanism is not clear. Therefore, blood sICAM-1 concentration increase may be due to several mechanisms:

- an increase in the shedding of the ICAM-1 due to increased secretases activaty
- an increase in the expression of ICAM-1 on cell membranes with no changes in secretases activity
- an increase both in the shedding of the ICAM-1 and in the expression of ICAM-1 on cell membranes.

If we suppose that the amount of ICAM-1 expressed on membranes (endothelium and leukocytes) remains relatively stable, an increase in the shedding of ICAM-1 will increase the circulating concentration of sICAM-1 and lower the possibilities of initiating leukocyte diapedesis. The regulation of sICAM-1 in blood is not clear. However, secretases responsible for the shedding may be stimulated through catecholamines and other factors. One study showed that exercise induced increase in sICAM-1 may be blocked by the infusion of propanolol (Rehman et al., 1997). Moreover, we have shown that catecholamines (especially noradrenaline) and diverse cytokines are produced and released in circulation after cold stimulation, especially after a 2-min exposure at −110°C (Leppäluoto et al., 2008). Therefore, cold exposure could induce an extra release of sICAM-1 and subsequently could induce a decrease of muscle inflammation.

A few years ago, we studied the effects of psychological and physical stress in humans on the concentration of many analytes and stress hormones, and among those compounds we analyzed serum sICAM-1 (Dugué et al., 1999). We observed an increased concentration of circulating sICAM-1 after both psychological (driving license examination) and thermal (sauna and bathing in ice-cold water) stress. Other authors have observed similar changes after cold exposure (Buemi et al., 1997).

Interestingly, in the thermal stress setting we had regular winter swimmers (some enthusiastic people appreciating a dip in ice-cold water several times per week during the winter season e.i. October to April in Finland where those studies were conducted) among our volunteers. At the end of the winter season, our regular winter swimmers had a higher concentration of sICAM-1 compared with occasional winter swimmers. Adaptation to repeated hot and cold stress has been postulated as a mechanism resulting in increased resistance to stress and diseases, and winter swimmers feel that regular swimming/bathing in ice-cold water is wholesome and that they fall ill less often than others (Dugué and Leppänen, 2000). Similarly, regular exercise is suppose to be wholesome. However, exercise training over a period of 2 months leads to an increase in sICAM-1 (Baum et al., 1994).

Nevertheless, there is for the time being no information available on the changes of sICAM-1 due to the combine effects of EIMD and whole-body cryostimulation (maybe a better term in that context than cryotherapy). sICAM-1 may be an important regulating substance in EIMD and cryostimulation and it should definitely be advisable to investigate that compound in that context.

## Conflict of interest statement

The author declares that the research was conducted in the absence of any commercial or financial relationships that could be construed as a potential conflict of interest.
